# Antimicrobial and Immunomodulatory Activities of PR-39 Derived Peptides

**DOI:** 10.1371/journal.pone.0095939

**Published:** 2014-04-22

**Authors:** Edwin J. A. Veldhuizen, Viktoria A. F. Schneider, Herfita Agustiandari, Albert van Dijk, Johanna L. M. Tjeerdsma-van Bokhoven, Floris J. Bikker, Henk P. Haagsman

**Affiliations:** 1 Department of Infectious Diseases and Immunology, Division of Molecular Host Defence, Faculty of Veterinary Medicine, Utrecht University, Utrecht, the Netherlands; 2 Department of Oral Biochemistry, Academic Centre for Dentistry Amsterdam, University of Amsterdam, VU University Amsterdam, Amsterdam, The Netherlands; University of Central Florida College of Medicine, United States of America

## Abstract

The porcine cathelicidin PR-39 is a host defence peptide that plays a pivotal role in the innate immune defence of the pig against infections. Besides direct antimicrobial activity, it is involved in immunomodulation, wound healing and several other biological processes. In this study, the antimicrobial- and immunomodulatory activity of PR-39, and N- and C-terminal derivatives of PR-39 were tested. PR-39 exhibited an unexpected broad antimicrobial spectrum including several Gram positive strains such as *Bacillus globigii* and *Enterococcus faecalis*. Of organisms tested, only *Staphylococcus aureus* was insensitive to PR-39. Truncation of PR-39 down to 15 (N-terminal) amino acids did not lead to major loss of activity, while peptides corresponding to the C-terminal part of PR-39 were hampered in their antimicrobial activity. However, shorter peptides were all much more sensitive to inhibition by salt. Active peptides induced ATP leakage and loss of membrane potential in *Bacillus globigii* and *Escherichia coli*, indicating a lytic mechanism of action for these peptides. Finally, only the mature peptide was able to induce IL-8 production in porcine macrophages, but some shorter peptides also had an effect on TNF-α production showing differential regulation of cytokine induction by PR-39 derived peptides. None of the active peptides showed high cytotoxicity highlighting the potential of these peptides for use as an alternative to antibiotics.

## Introduction

Short cationic amphiphilic peptides have attracted considerable attention in the past years by both scientist as pharmacists due to their natural antimicrobial properties and the ability to modulate the immune responses of the host. These so-called host defence peptides (HDPs) are ubiquitously present in all classes of life and serve as the first line of defence against bacterial, fungal, and viral infections [1]. With few exceptions, HDPs are amphipathic (spatial separation of hydrophilic and hydrophobic residues), positively charged, and contain a high number of hydrophobic residues [2]. Based on their molecular properties and structural conformations, HDPs can be divided into several classes of which cathelicidins and defensins are the largest and best described [3].

The pig has a large reservoir of cathelicidins relative to other mammals [4]. Based on their primary amino acid compositions, porcine cathelicidins fall into three subgroups: linear proline-rich cathelicidins (PR-39, Prophenin 1 and 2), disulfide-rich protegrins 1–5, and a arginine/histidine rich myeloid subgroup [5]. PR-39 was originally isolated from porcine small intestine [6], but subsequent cDNA cloning showed that PR-39 is also expressed in bone marrow [7] and neutrophils [8]. PR-39 is secreted as a prepropeptide that undergoes post-translational modification by the cleavage of the N-terminal portion releasing the mature form of 39 C-terminal amino acids [9]. This mature PR-39 is active against a broad spectrum of bacteria, including multidrug resistant clinical isolates [10]–[12]. Similar to other proline-rich peptides, PR-39 does not only promote cell lysis by membrane perturbation, but translocates across the membrane and disrupts various cellular processes such as DNA and protein synthesis [13]. Besides its antimicrobial properties, PR-39 has been shown-to induce migration of neutrophils in a calcium dependent manner [14], to modulate macrophage viability by inhibiting apoptosis [15], and to function as an anti-apoptotic factor in endothelial cells during hypoxia [16]. Many other biological processes such as regulation of angiogenesis [17], promotion of wound repair [18], [19], and prevention of inflammation during tissue injury [18] have also been reported. The antimicrobial potential of PR-39 *in vivo* was elegantly demonstrated in a study where transgenic mice, expressing PR-39, were protected against Group A *Streptococcus* compared to the control group [20], although it is not clear whether this was achieved through direct or indirect effects of PR-39 on bacterial viability and virulence.

In this study we seek to find core elements of PR-39 involved in antimicrobial activity and immunomodulation. For this purpose, we synthesized N- and C terminally truncated variants of PR-39 and determined their antimicrobial activities, cytotoxicity effects, and the ability to modulate the IL-8 and TNF-α response of porcine macrophages.

## Materials and Methods

### Mammalian Cell lines

Porcine intestinal epithelial IPEC-J2 [21] and porcine alveolar macrophage 3D4/31 (American Type Culture Collection; ATCC-CRL-2844) were the two cell lines used throughout this study. Cells were cultured and maintained in advanced DMEM/F12 medium (Gibco; supplemented with 5% Fetal Calf Serum (FCS; vol/vol), 10 U/ml penicillin, 10 mg/ml streptomycin, 2 mM L-glutamine) and ATCC RPMI-1640 medium (supplemented with 10% FCS (vol/vol), 1% non-essential amino acids (Gibco), respectively. Both cell lines were grown at 37°C under 5% CO_2_+95% air condition. The medium was changed every other day until the cells reached 80% confluence prior to the next passage. The culture handling and sub-culture procedures for the 3D4/31 cell line were carried out according to the protocol provided by the distributor.

### Bacterial strains

Four *Escherichia coli* strains (ATCC 25922, ATCC 4157, K12 and K88), 2 *Bacillus globigii* strains (BM013; and ATCC 6633), *Bacillus licheniformis* (ATCC 21424), *Bacillus cereus* (ATCC 9193), *Streptococcus pyogenes* (ATCC19616); *Enterococcus faecalis* (ATCC 29213), *Enterococcus faeceum* E155, *Staphylococcus aureus* (ATCC 29213) and MRSA (WKZ-2, human isolate) bacterial strains were all cultured in TSB at 37°C (Tryptone Soy Broth; Oxoid).

### Peptide synthesis

Peptides corresponding to C- and N-terminal domains of PR-39 were synthesized using Fmoc solid-phase synthesis as described previously [22]. All peptides were purified to a minimum purity of 95% by reverse phase high-performance liquid chromatography prior to biological testing. The sequences of the peptides used in this study are shown in Table 1.

**Table 1:** Activity of PR-39 derived peptides against *B. globigii* and *E. coli*.

				MBC (µM)			
				*E. coli*		*B. globigii*	
Peptide	Amino acid sequence	Length	Charge	0 mM NaCl	100 mM NaCl	0 mM NaCl	+100 mM NaCl
PR-39	RRRPRPPYLPRPRPPPFFPPRLPPRIPPGFPPRFPPRFP	39	+10	1.25	2.5	2.5	5
PR-39(1–26)	RRRPRPPYLPRPRPPPFFPPRLPPRI	26	+8	2.5	1.25	5	5
PR-39(1–22)	RRRPRPPYLPRPRPPPFFPPRL	22	+7	1.25	2.5	2.5	10
PR-39(1–18)	RRRPRPPYLPRPRPPPFF	18	+6	5	20	2.5	10
PR-39(1–15)	RRRPRPPYLPRPRPP	15	+6	5	>40	5	>40
PR-39(16–39)	PFFPPRLPPRIPPGFPPRFPPRFP	24	+4	10	>40	10	>40
PR-39(20–39)	PRLPPRIPPGFPPRFPPRFP	20	+4	5	>40	20	>40
PR-39(24–39)	PRIPPGFPPRFPPRFP	16	+3	40	>40	40	>40

**Figure 1:**
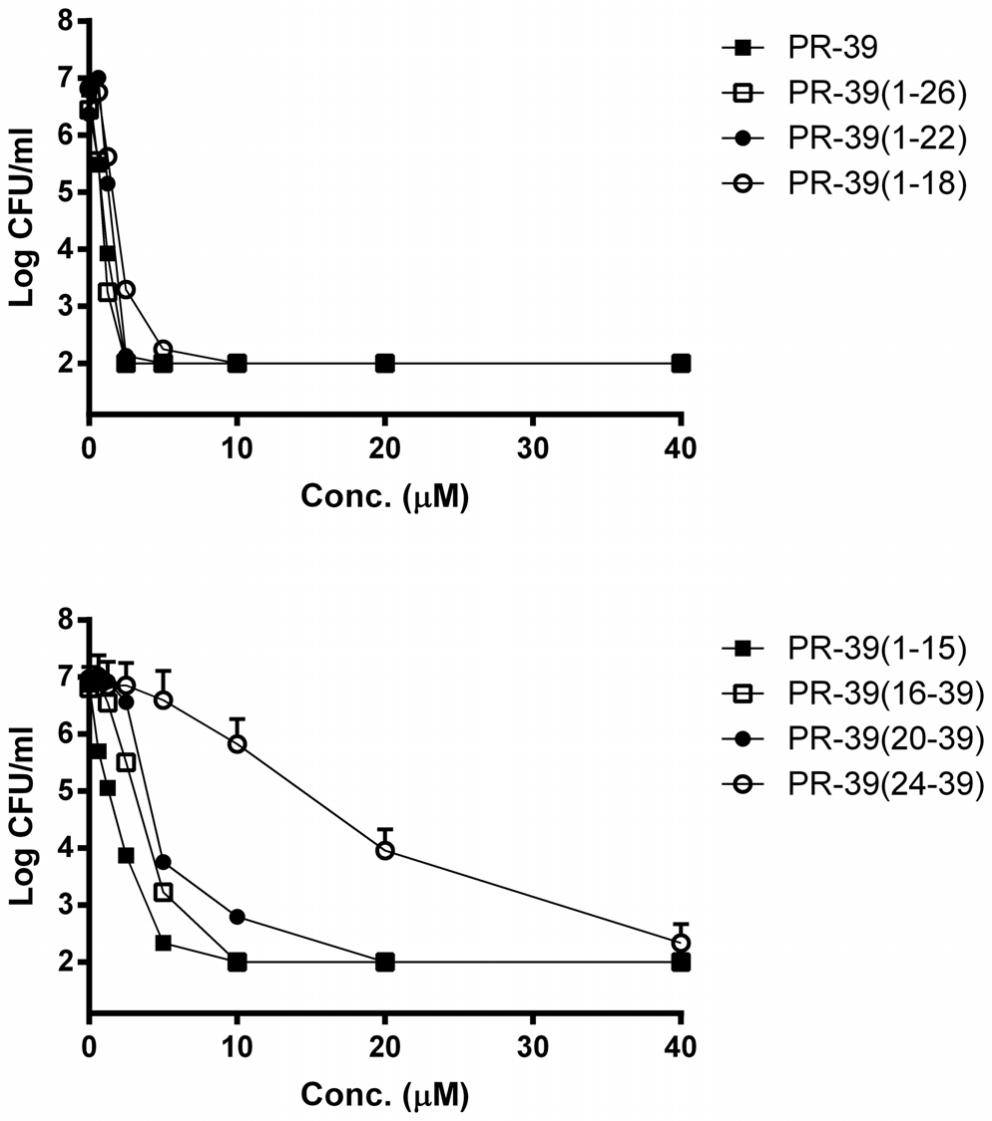
PR-39 derived peptides are active against *Bacillus globigii*. Peptides were incubated for 3×10^6^ CFU/ml *B. globigii* in phosphate buffer (10 mM, pH 7; 1/100 TSB). Bacteria were serially diluted, plated on TSA plates and counted after 24 h. Shown are mean ± SEM of n≥3. For clarity, the data of 8 peptides is divided over 2 figures.

Colony counting assays were performed as in Fig 1. MBC was determined with or without addition of 100 mM NaCl to the buffer.

### Antimicrobial Activity Assays

#### Broth dilution assays

Initial screening for antibacterial activity of PR-39 was performed using broth dilution assays. Bacteria were grown to mid-logarithmic phase in MHB medium. The optical density was measured and bacteria were diluted to 2×10^6^ CFU/ml. Subsequently 25 µl bacteria were incubated with 25 µl peptide in polypropylene 96 wells plates and incubated at 37°C. After 3 h 200 µl MHD medium was added and samples were further incubated overnight. Minimal inhibitory concentrations (MIC) were determined by turbidity measurement of the wells.

#### Colony counting assays

Overnight cultures of *E. coli* and *B. globigii* were grown in TSB medium at 37°C to mid-logarithmic phase. The antimicrobial activity of the PR-39 derived peptides was tested using colony counting as described before [23]. In short, cells were collected via centrifugation at 900×g for 10 minutes, and resuspended in 10 mM phosphate buffer, pH 7.0, containing 1/1000 TSB medium (Buffer A). Cells were further diluted to 2×10^6^ CFU/ml in the same buffer. Next, 25 µl of cell suspension was mixed with an equal volume of different concentrations of peptides ranging from 0–80 µM, and incubated further for 3 hours at 37°C. Ten-fold dilutions were prepared in minimal buffer containing 1/1000 TSB medium in distilled water, and 100 µl from each dilution was spread on TSA plates (Trypton Soy Agar; Oxoid). The plates were incubated at 37°C and the number of colonies was counted 24 h later to determine the number of surviving bacteria. Minimal Bactericidal Concentration (MBC) was defined as <100 CFU/ml corresponding to the detection limit of this assay. To determine the dependence of activity of PR-39 on the energy status of the cell, *E. coli* was incubated 30 min at 37°C in the presence of the metabolic uncouplers 50 µM carbonyl cyanide m-chlorophenylhydrazone (CCCP), or 5 mM 2,4-dinitrophenol (DNP). Subsequently, an antimicrobial activity assay was performed as described above.

### ATP leakage measurements

*B. globigii* and *E. coli* were grown in TSB medium at 37°C to mid-logarithmic phase. Bacteria were centrifuged, resuspended in buffer A and diluted to 2×10^7^ CFU/ml. Sixty µl bacteria were incubated with 60 µl 0.5 or 3 µM peptide for 5 min at 37°C. The samples were centrifuged, supernatant was stored at 4°C until further use and the bacterial pellet was resuspended in boiling 100 mM Tris, 4 mM EDTA and further incubated at 100°C to lyse the cells. The lysed cells were centrifuged and the supernatant was kept on ice. Subsequently, both intra- and extracellular ATP levels were determined using the Roche ATP bioluminescence kit CSI II (Roche Diagnostics Nederland B.V., Almere, the Netherlands), according to the manufacturer's protocols.

### Determination of membrane potential

*B. globigii* was grown in TSB medium at 37°C to mid-logarithmic phase. Bacteria were pelleted and redissolved in buffer A to a density of 5×10^7^ CFU/ml. To 2 ml of bacterial culture 20 µl 0.15 mM DiSC3(5) (3,3′ - Dipropylthiadicarbocyanine iodide, Life Technologies, Europe BV, the Netherlands) and 50 µl 1 M glucose were added until a stable fluorescence signal was reached. Peptide was added from a 1600 µM stock solution (final concentration depending on the peptide used) and fluorescence was continuously monitored (excitation 640 nm, emission 670 nm, slits 10 nM).

### Cytotoxicity Assay

The cytotoxic effect of PR-39 derived peptides on porcine intestinal epithelial IPEC-J2 and porcine macrophage 3D4/31 cells was determined using the cell proliferation reagent WST-1 (Roche), which measures cell viability based on glycolytic production of NAD(P)H, as described by the manufacturer. Approximately 5×10^4^ cells were seeded into a 96-well microtiter plate and were incubated at 37°C with 5% CO_2_ until an 80% confluent monolayer was reached. Next the media were replaced with 50 µl of fresh media containing peptides to a final concentration ranging from 0–40 µM/well. After 24 hours incubation at 37°C, the old peptide-containing medium was removed, and 100 µl of fresh medium containing 10% WST-1 reagent was added to each well, followed by 30 min incubation at 37°C in the dark. Subsequently, the absorbance was measured at 450 nm using 650 nm as the reference wavelength.

### Peptide induced IL-8 and TNF-α production

The ability of mature PR-39 and PR-39 derived peptides to modulate cytokine production in macrophage 3D4/31 cells was measured by means of an enzyme-linked immunosorbent assay (ELISA). Cells (5×10^4^ cells) were seeded into 96-wells microtiter plates and grown to an 80% confluent monolayer prior to the assessment. After 24 h the culture medium was replaced with fresh culture medium to remove non-adherent cells prior to peptide stimulation. On the next day the culture medium was discarded from each well and 100 µl of fresh medium containing 0 or 20 µM of peptides tested was added to the cells and incubation was continued at 37°C. The culture supernatants were collected at 4 and 24 h time points. The expression levels of porcine IL-8 and TNF-α were measured using the commercial available DuoSet ELISA kits (R&D Systems), following the protocols provided by the manufacturer. All samples were centrifuged briefly at 5000 rpm for 3 minutes at room temperature to remove cell debris prior to use. The microtiter plates were read at an absorbance of 450 using 550 nm as a reference wavelength to correct for optical imperfections of the microtiter plate.

### Statistics

Statistical analysis of variance was performed using SPSS Version 20 for windows. All data were analysed by one way ANOVA with a Bonferroni multi comparison post-hoc test. Significant differences between means were defined as p<0.05.

## Results

### Antimicrobial activity of PR-39 derived peptides

In order to determine the antibacterial spectrum of full length PR-39, an initial screening was performed on multiple strains using broth dilution assays in 50% MHB medium. Except for both *S. aureus* strains that were resistant to PR-39 at the tested concentrations, all other Gram positive strains were as susceptible to PR-39 as *E. coli* (Table S1). To obtain a more detailed profile of the antimicrobial potency, mature PR-39 and PR-39 derived peptides were tested by means of colony counting assay against *E. coli* and *B. globigii*, (Fig. 1, Table 1). Only minor differences were observed between the two bacterial strains with respect to susceptibility to the PR-39 derived peptides. The MBC of mature PR-39 for *E. coli* was 1.25 µM while it was slightly higher for *B. globigii* at 2.5 µM. The truncated peptides were all antibacterial but with some differences in activity. N-terminal peptides PR-39(1–26), PR-39(1–22), PR-39(1–18) and PR-39(1–15) had activities close to mature PR-39, with some small increases in MBC values for the shorter peptides. C-terminal peptides all had reduced activity compared to the mature peptide, but only PR-39(24–39) had a severely reduced activity with complete killing of bacteria only at 40 µM. These results indicate that the N-terminus of PR-39 is contributing mostly but not exclusively, to the antibacterial activity of PR-39.

### Effect of salt and energy status of the cell on antimicrobial activities of PR-39 derived peptides

The effect of salt on the antibacterial activities of the peptides was evaluated by the addition of 100 mM NaCl to the reaction buffer. The mature peptide was hardly affected by the higher ionic concentration, but the short PR-39(1–15) peptide showed highly reduced antimicrobial activity against *E. coli* and *B. globigii* (Table 1). In addition, loss of functionality was also apparent for the C-terminal peptides *e.g*. PR-39(16–39), PR-39(20–39), PR-39(24–39). This result indicates a correlation between salt-induced inhibition of antimicrobial activity and total charge (but not charge density) of the peptide. The effect of the metabolic uncouplers CCCP and DNP on the antimicrobial activity of PR-39 was also determined. Incubation of bacteria with 5 mM DNP or 50 µM CCCP resulted in an inhibition of growth of *E .coli* (showing the effectiveness of the uncouplers), but did not lead to a changed susceptibility towards PR-39 (data not shown). This indicates that bacterial energy dependant uptake of peptide is not required for PR-39's activity.

### ATP leakage

**Figure 2:**
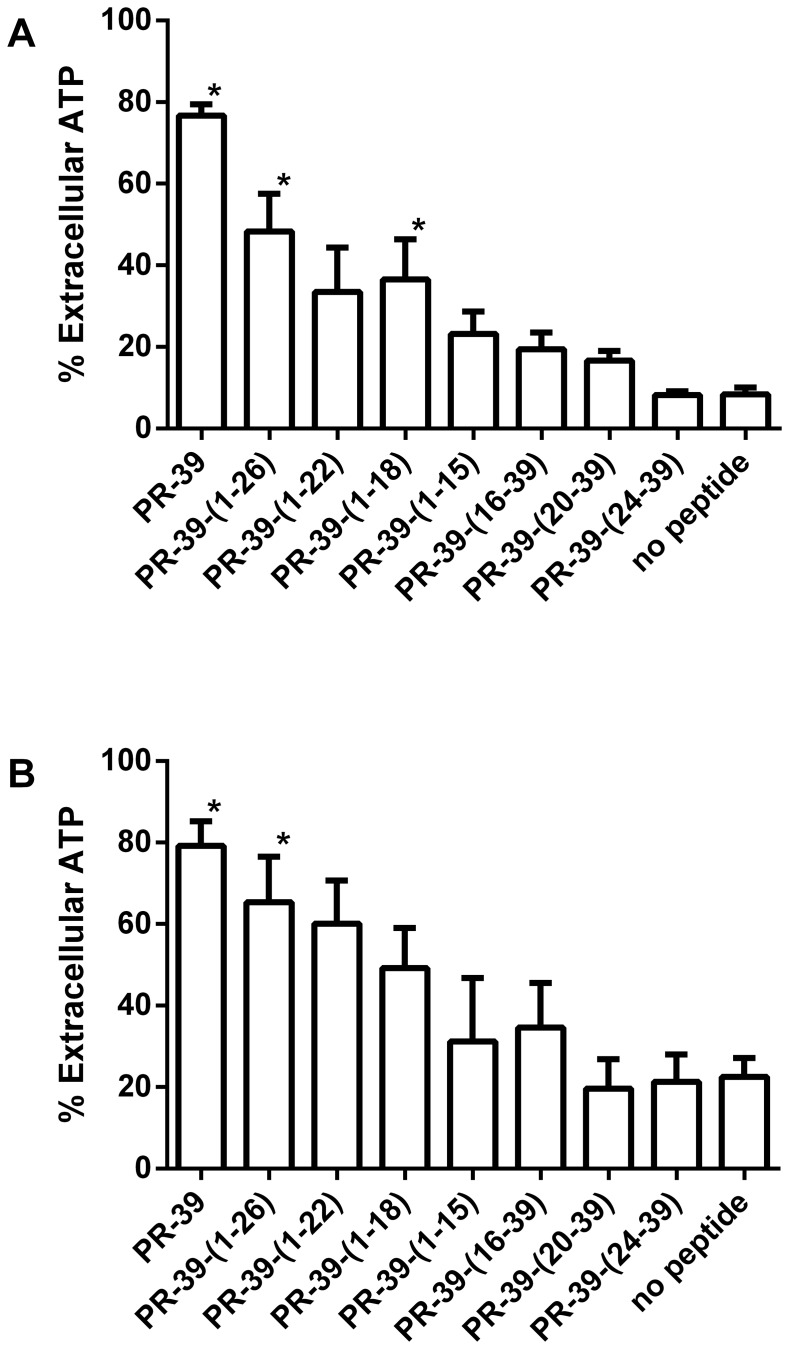
PR-39 derived peptides cause release of bacterial ATP. Bacteria were incubated for 5 µM peptides. ATP in supernatant and cell pellet were determined using a luciferase bioluminescence kit. **A**: *E. coli*, **B**: *B. globigii*. Shown are mean ± SEM of at least three independent experiments in triplicate. *: p<0.05 compared to the no-peptide control.

A 5 min incubation of bacteria with 3 µM PR-39 and derived peptides resulted in a substantial loss of ATP from bacterial cells (Fig. 2). Full length PR-39 exhibited the largest effect leading to approx. 80% extracellular ATP in both *E. coli* and *B. globigii*. ATP loss largely followed the antimicrobial activity of the peptides with low effects of C-terminal peptides and also smaller effects for shorter N-terminal peptides. Incubation of bacteria (both *E. coli* and *B. globigii*) with 0.5 µM PR-39 derived peptides did not have a significant effect on ATP release for any of the peptides (data not shown).

### Membrane potential

**Figure 3:**
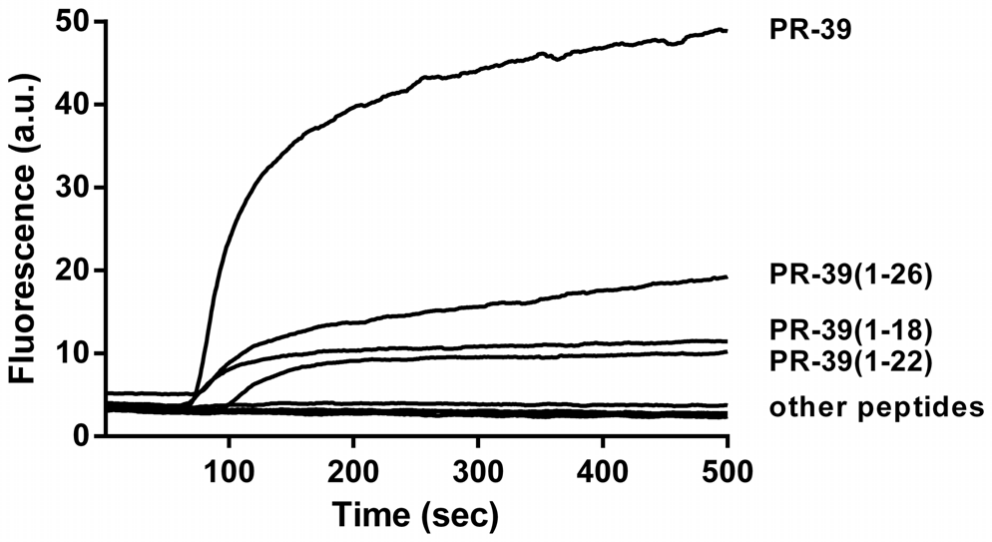
PR-39 is most active in disrupting the membrane potential of *B. globigii*. The membrane potential sensitive dye DiSC3(5) was incubated with bacteria until a stable baseline was formed. Fluorescence increase upon addition of peptide was measured at Excitation/Emmission 640/670 nm. Shown are representative curves of:PR-39: 2.5 µM, PR-39(1–26): 2.5 µM; PR-39(1–22): 2.5 µM; PR-39(1–18): 2.5 µM; PR-39(1–15): 5 µM; PR-39(16–39): 10 µM; PR-39(20–39): 20 µM; PR-39 (24–39): 10 µM.

The effect of peptides on the membrane potential was measured by monitoring the membrane potential using the fluorescent dye DISC3(5) (Fig. 3). Peptides were tested against *B. globigii* at concentrations close to the determined MBC values for each peptide in the buffer solution used. PR-39 (2.5 µM) caused a large and immediate increase in fluorescence (indicative of loss of membrane potential) that continued to slowly increase in the following minutes. Shorter N-terminal peptides showed a smaller effect, while C-terminal peptides and PR-39(1–15) did not have an effect. Viability assays after the measurement indicated that all samples contained >10^6^ CFU/ml except for PR-39 which had an average of 3.10^5^ CFU/ml indicating that less bacteria were viable after incubation with the mature peptide. At lower concentrations (1 µM) no effect on membrane potential was seen for any of the peptides.

### Cytotoxicity effects of PR-39 derived peptides

**Figure 4:**
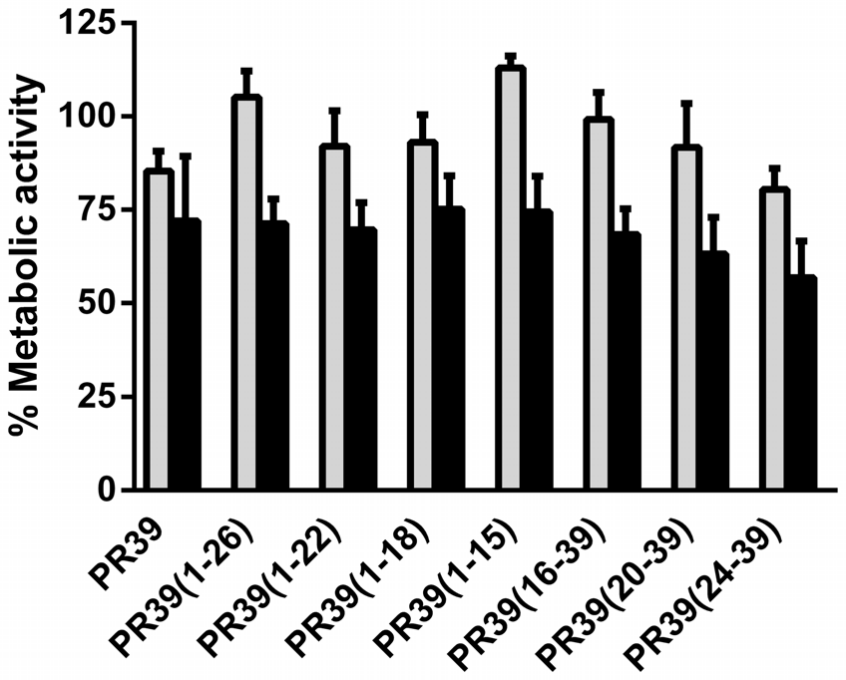
PR-39 derived peptides have low cytotoxicity. Porcine macrophages (3D4/31 cells) were incubated for 24 h with 0–40 µM peptide. Metabolic activity was determined using WST-1 reagent. For clarity, the metabolic activity compared to the control (no peptide, 100%) is shown only for 5 (grey) and 40 µM (black) peptide. Shown are mean ± SEM of at least three independent experiments. For full data set please see Table S2.

The toxic effect of PR-39 derived peptides towards alveolar macrophage 3D4/31 cells was assayed after a 24 h exposure at 37°C (Fig. 4). A relatively small reduction (p>0.05) in metabolic activity of these cells was seen upon addition of PR-39 derived peptides. At 40 µM, a concentration much higher than where PR-39 exerts its antibacterial effects, PR-39 lowered the metabolic activity to approximately 70% compared to non-treated control 3D4/31 cells. The same concentration of the other PR-39 derived peptides had a similar effect with only small changes in magnitude of the reduction, all not reaching statistical significance from the control. Similar results were obtained for porcine intestinal epithelial cells (IPEC-J2, Table S2), indicating that the low cytotoxic effect is a general characteristic of these peptides.

### Induction of IL-8 and TNF-α production by PR-39 derived peptides

**Figure 5:**
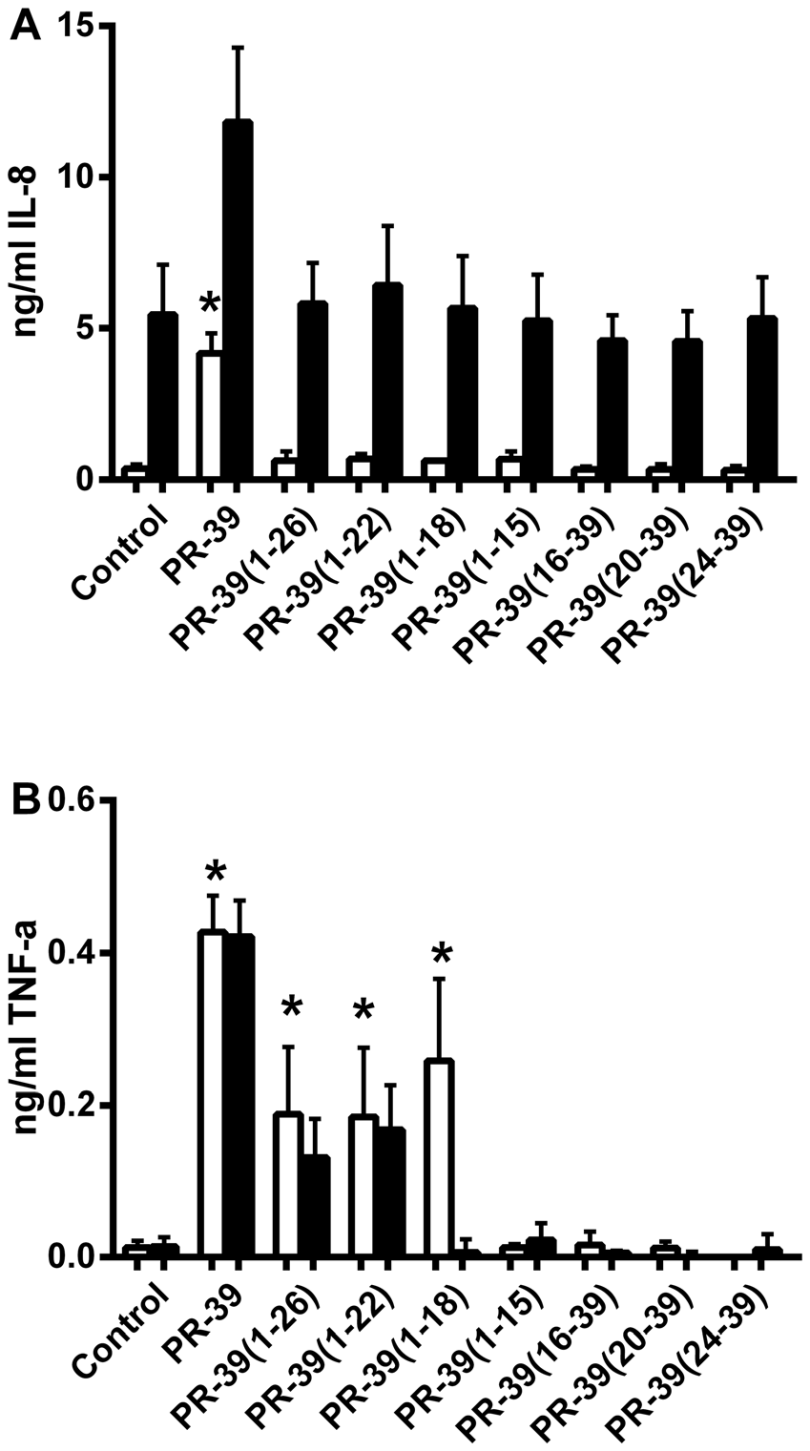
PR-39, but not shorter PR-39 derived peptides induce IL-8 production. Porcine macrophages (3D4/31 cells) were incubated with 20 µM peptides for 4 (white bars) or 24 h (black bars). A) IL-8 and B) TNF-α production in the cell supernatant was determined using ELISA. Shown are mean ± SEM of at least three independent experiments. *: p<0.05 compared to the no peptide control.

Besides antibacterial effects, HDPs are known to have immunomodulatory effects on host cells. The effect of PR-39 derived peptides on IL-8 and TNF-α production in 3D4/31 cells was investigated. After 4 h incubation a significant 10-fold increase in IL-8 production was observed for the mature PR-39 peptide compared to the control (Fig. 5). PR-39 derived peptides did not induce a significant increase. After 24 h of incubation, only PR-39 induced IL-8 production while all other peptides did not. Interestingly, the IL-8 level after 24 h of the control samples increased to 4 ng/ml, indicating that the cells produced IL-8 under these conditions. PR-39 also induced TNF-α in 3D4/31 cells although to a 10-fold lower extent than IL-8. TNF- α induction was not limited to the full size peptide because some of the N-terminal peptides induced similar amounts of TNF- α C-terminal peptides did not have any effect on TNF-α production indicating that the core element of the peptide required for this activity lies in the N-terminal part of the protein.

## Discussion

Current literature on PR-39 has shifted from an initial focus on antibacterial activity to potential new roles in host defence mechanisms. In the search for new peptide antibiotics, PR-39 is, due to its high proline content, an excellent lead compound showing high stability in solution. PR-39 is resistant to serine proteases, elastase, and aminopeptidases, which results in a long half-life [5]. This could be an important feature if PR-39, or peptides derived thereof, are used for therapeutic purposes.

It has previously been demonstrated that PR-39 exhibits a broad antibacterial spectrum against Gram-negative bacteria excluding *Pseudomonas*, which has been described as insensitive towards PR-39 [24]. Although Gram positive strains are considered less susceptible, our study showed that 7 Gram-positive strains, including 4 *Bacillus* strains, were all susceptible to PR-39. Only *S. aureus* showed resistance against PR-39 *in vitro*. This observation, showing that antibacterial activity of PR-39 is not completely restricted to Gram-negative bacteria is supported by only a small number of reports [25], [26].

PR-39 is a member of a large family of proline rich antimicrobial peptides (PR-AMPs). Mammalian PR-AMPs include the well-studied bovine Bac5 and Bac7 from bovine neutrophils and OABac11 and OABac6 from sheep. Many more members of the same family are found in for example insects and amphibians. For most of these members only antimicrobial activities have been determined, interactions with host cells as described for PR-39 have not been extensively studied. More detailed information on the activity of the family of PR-AMPs can be found in an excellent review by Scocchi *et al* [27]. In general, most PR-AMPs are active against Gram-negative bacteria, indicating a comparable mechanism of action of all peptides within this group. Several short PR-AMPs for example, apidaecins and pyrrhocoricins from honeybee and fire bug, respectively [28], [29] have comparable activity and specificity compared to mammalian PR-AMPs. This is largely in line with the observations in this report that short PR-39 fragments are also antimicrobial.

Several reports using PR-39(1–15) and PR-39(1–26) peptides have stated that the antibacterial activity of PR-39 is located in the N-terminus of the peptide. More notably, the positive charge of the first 3 amino acids of PR-39, as well as Leu9 and its following amino acids (PRPR) were found essential for full activity [25], [26]. PR39(1–26) was described as slightly more active than full length PR-39 while antibacterial activities of PR-39(1–15) and PR-39 were similar, although a direct comparison between the 2 peptides was only performed with one single strain; a PhoP- mutant of *Salmonella typhimurium* [30]. Our results confirm these previous observations, showing relatively small differences in MBC between the 5 N-terminal peptides, and extend these observations for activity against *B. globigii*. In addition, our results showed that C-terminal peptides indeed have lower activities than N-terminal peptides but that peptides lacking the ‘essential’ N-terminal amino acids, such as peptides PR-39(16–39) and PR-39(20–39) still have considerable antibacterial activity. Finally, the effect of ionic strength on the activity of peptides indicate that one has to be careful using MIC values as indicators for antimicrobial activity. The activity of full length PR-39 was hardly affected by 100 mM NaCl while shorter peptides lost most of theirs. These effects are likely due to reduction of the initial electrostatic interaction between negatively charged bacterial membranes and positively charged peptides. Based on our data, the inhibiting effect seems to be related more to total charge of the peptide instead of charge density since PR-39(1–15) has a higher charge/length ratio than other peptides, yet still is inhibited strongly by salt.

The exact mechanism of PR-39's antibacterial activity is currently not exactly understood but it is generally thought that PR-39 and other PR-AMPs kill bacteria by a non-lytic mechanism [27]. For PR-39, this non-lytic mechanism was mainly based on original observations made by Boman *et al*, who noticed a lag-time between interaction of peptide with bacteria and actual killing [13]. More recently, it was shown that the activity of PR-AMPs, including PR-39 was reduced when mutations were formed in the *sbmA* gene of *E. coli* [31], [32]. This gene is predicted to encode a component of an inner membrane transporter belonging to the ATP-binding-cassette superfamily of transporter proteins. It is hypothesized that this transporter is used to translocate PR-39 over the bacterial membrane. In support of this hypothesis, it was shown for Bac7, that the peptide was inactive against *E. coli* and *S. enteritidis* when the ATP-dependent transporter was inactivated through the use of the metabolic uncoupler DNP [33]. Interestingly, *smbA* has been identified in several Gram-negative bacteria but not in *Pseudomonas aeruginosa*, a bacterial strain relatively resistant to PR-AMPs. In addition *smbA* has not been described in Gram-positive bacteria, which are generally also considered less susceptible to PR-39 and other PR-AMPs [32].

After energy dependent uptake, PR-AMPs bind to DnaK as their intracellular target [34]. DnaK belongs to the HSP70 family of chaperone proteins and binding of peptide interferes with normal protein folding in bacterial cells. For oncocin, an activity-optimized PR-AMP, it was shown that it binds with its N-terminal residues PPYLPR (AA 4–9) to the substrate binding site of DnaK [35]. Interestingly PR-39's N-terminal sequence contains an identical motif at the N-terminus (AA 6–11) indicating that it would bind similarly to DnaK. However, despite the binding evidence pointing towards a DnaK inhibiting function of PR-AMPs, DnaK deficient *E. coli* was found equally susceptible as wild type *E. coli* to Bac7(1–35) [27]. This indicates that other unidentified (intracellular) targets apart from DnaK could be involved in the bactericidal mode of action of PR-AMPs.

In our experimental set-up, we observed some contrasting results to the general hypothesis on the antimicrobial working mechanism of PR-39. Firstly, our PR-39 peptides are as active against Gram positive strains (except *S. aureus*) as against Gram negative bacteria. In addition we did not observe an effect of metabolic uncouplers DNP or CCCP on PR-39 activity, and finally we showed fast ATP leakage and loss of membrane potential upon incubation with PR-39. These data are more in line with a lytic mechanism or at least membrane perturbing mechanism of PR-39 than with a mechanism where ATP-dependant uptake of peptide is required. Interestingly, a lytic mechanism was also proposed by Vunnam *et al* based on clearance of an existing monolayer of *B. subtilis* on agar treated with PR-39 [24]. In addition, the D-enantiomer was at least as active as the all-L isoform of PR-39 also indicating a non-chirality dependant mechanism of action for this peptide. For Bac7 it was shown that the peptide actually had a dual mode of action, energy and uptake dependant at sub-MIC concentrations, while a lytic mechanism was observed at higher concentrations [33]. At the moment it is unclear what causes the discrepancy in observed mode of action (lytic vs intracellular uptake) between the current study and earlier described experiments. However, the observed specific resistance of *S. aureus* instead of all Gram positive strains towards PR-39, could possibly be explained by the presence of *S. aureus* specific proteases that cleave PR-AMPs, despite their proposed insensitivity towards proteases. The main indication for this hypothesis is the fact that *S. aureus* is very susceptible to the D-isomer of PR-39 [24]. *S. aureus* is known to produce several proteases which helps it to evade the immune system. For example, the secreted proteases aurelysin, V8 and Staphopain A &B are all involved in evasion of complement [36]. Another indication that protease activity could affect susceptibility towards PR-AMPs was recently described [37]. In this study, *E. coli* oligopeptidase effectively cleaved several PR-AMPs and overexpression of this protease resulted in increased resistance towards PR-AMPs. It would be very interesting to determine further the susceptibility of PR-39 to *S. aureus* specific- and other bacterial proteases.

The cytotoxicity measured for PR-39 and its derivatives was low towards porcine macrophages (3D4/31 cells) and intestinal cells (IPEC-J2), although at concentrations well above the MIC values some reduction in metabolic activity was observed. The low cytotoxicity for PR-39 is in agreement with the general feature of low cytotoxicity found in many PR-AMPs [32]. Low uptake of peptide by mammalian cells and lack of binding to mammalian HSP70 proteins are considered the main reason for this. However cytotoxicity of PR-39 seems to be cell specific, as was shown by Catrina *et al* who tested several different (mouse and human) cells [38]. Interestingly, using labelled PR-39 peptide they showed that cytotoxicity of PR-39 was related to the intracellular localization of the peptide indicating that only cells with an uptake mechanism suitable for PR-39 were affected. A similar cell dependency was observed for highly susceptible rat intestinal cells compared to MDCK cells. In this latter study PR-39(1–26) was also tested, which had a much lower cytotoxicity than the mature peptide, but no data on peptide uptake were shown.

Immunomodulatory activity of PR-39 derived peptides was determined by means of measuring expression of IL-8 and TNF-α in porcine macrophages upon peptide incubation. With respect to IL-8 only full length PR-39 was capable of inducing expression of this cytokine. However, besides full length PR-39 also some N-terminal derivatives were capable to induce TNF-α production, although to a lower extent than Il-8. This indicates that signalling cascades leading to TNF- α and IL-8 expression are not necessarily linked in this porcine macrophage cell line. The mature peptide and PR-39(1–26) were described to have chemotactic properties towards neutrophils (but not mononuclear cells) [14], but possibly the mature peptide also induces chemotaxis indirectly via the upregulation of IL-8 in macrophages. Some other studies showed immunomodulatory effects of PR-39. Delfino *et al* described protection by PR-39 treatment against septic shock in mice. It was thought that PR-39 has a dampening effect on TNF-α levels which resulted in septic shock [39]. The lack of effect in our macrophage stimulation model of the shorter peptides indicates that the full sequence is required for this stimulation. Recently, it was described that oncocin and apidaecin, two short (<20 Amino acids) PR-AMPs also lack any immunomodulatory activity. No chemotactic activity towards DC, no modification of LPS induced immune responses or direct immune stimulating effects on macrophages were observed for these PR-AMPs, in contrast to the murine cathelicidin CRAMP used in the same study [40]. Taken together, our results show that N-terminal PR-39 derived peptides are sufficient for antimicrobial activity and stimulation of TNF-α production by macrophages, but that the full length peptide is required for IL-8 production.

## Supporting Information

Table S1 **Antibacterial activity of PR-39 against selected Gram positive and Gram negative bacteria.** Broth dilutions assays were performed to determine susceptibility of an array of Gram positive and Gram negative bacteria. Experiments were performed in triplicate and the range of MIC values is depicted.
(DOC)

Table S2 **Cytotoxicity of PR-39 towards intestinal and macrophage cells.** Intestinal and macrophage cells were grown to 80% confluency and incubated with PR-39 derived peptides at different concentrations for 24 h. Experiments were performed in triplicate and metabolic activity, measured is expressed in % relative to the untreated cell.
(DOCX)
