# Supplementary material for: Antimicrobial and Immunomodulatory Activities of PR-39 Derived Peptides
Source: PLoS One. 2014 Apr 22;9(4):e95939. doi: 10.1371/journal.pone.0095939 (PMC3995882; doi:10.1371/journal.pone.0095939)
Supplement: Table S1 — Antibacterial activity of PR-39 against selected Gram positive and Gram negative bacteria. Broth dilutions assays were performed to determine susceptibility of an array of Gram positive and Gram negative bacteria. Experiments were performed in triplicate and the range of MIC values is depicted. (DOC) [file pone.0095939.s001.doc]

**Table S1. Antibacterial activity of PR-39 against selected Gram positive and Gram negative bacteria.**

| **Bacterial strain** | **MIC (μM)** |
| --- | --- |
| *Bacillus* *Subtilis* (ATCC6633) | 2.5-5 |
| *Bacillus* Globigii (TNO) | 1.25-5 |
| *Bacillus* *Cereus* ATCC 9193 | 5-20 |
| *Bacillus* Lichen ATCC 21424 | 5 |
| MRSA (human clinical isolate) | >40 |
| *S. aureus* ATCC 29213 | >40 |
| *E.coli* ATCC25922) | 2.5-10 |
| *E.coli* K88 403 | 1.25-2.5 |
| *E.coli* K12 | 1.25-2.5 |
| *E.coli* ATCC 4157 | 1.25-2.5 |
| *Streptococcus Pyogenes* (ATCC 19616) | 2.5-5 |
| *Enterococcus Faecalis* (ATCC 29213) | 10 |
| *Enterococcus* *Faeceum* E155 | 1.25-2.5 |
