# Supplementary material for: Antimicrobial and Immunomodulatory Activities of PR-39 Derived Peptides
Source: PLoS One. 2014 Apr 22;9(4):e95939. doi: 10.1371/journal.pone.0095939 (PMC3995882; doi:10.1371/journal.pone.0095939)
Supplement: Table S2 — Cytotoxicity of PR-39 towards intestinal and macrophage cells. Intestinal and macrophage cells were grown to 80% confluency and incubated with PR-39 derived peptides at different concentrations for 24 h. Experiments were performed in triplicate and metabolic activity, measured is expressed in % relative to the untreated cell. (DOCX) [file pone.0095939.s002.docx]

**Table S2. Cytotoxicity of PR-39 towards intestinal and macrophage cells.**

| **IPEC-J2 cells** |  |  |  |  |  |  |  |
| --- | --- | --- | --- | --- | --- | --- | --- |
|  | **µM peptide** | | | | | | |
|  | **0.6** | **1.2** | **2.5** | **5** | **10** | **20** | **40** |
| PR-39 | 82 ± 4 | 81 ± 7 | 83 ±8 | 82 ± 12 | 74 ± 12 | 80 ± 16 | 68 ± 18 |
| PR-39(1-26) | 88 ± 8 | 94 ± 11 | 94 ± 11 | 93 ± 17 | 110 ± 18 | 88 ± 15 | 88 ±24 |
| PR-39(1-22) | 105 ± 12 | 113 ± 15 | 96 ± 16 | 97 ± 14 | 113± 22 | 101 ± 18 | 105 ± 15 |
| PR-39(1-18) | 115± 17 | 100 ± 7 | 115 ± 16 | 106 ± 15 | 111 ± 19 | 96 ± 10 | 90 ± 11 |
| PR-39(1-15) | 110 ± 12 | 107 ± 15 | 121 ± 15 | 103 ± 9 | 118 ± 15 | 119 ± 21 | 107 ± 9 |
| PR-39(16-39) | 112 ± 11 | 122 ± 11 | 123 ± 13 | 121 ± 15 | 107 ± 1 | 128 ± 6 | 109 ± 12 |
| PR-39(20-39) | 114 ± 16 | 116 ± 8 | 125 ± 18 | 119 ± 13 | 126 ± 11 | 122 ±13 | 107 ± 8 |
| PR-39(24-39) | 115 ± 16 | 107 ± 14 | 111 ± 11 | 108 ± 8 | 114 ± 16 | 117 ± 8 | 117 ± 8 |
|  |  |  |  |  |  |  |  |
|  |  |  |  |  |  |  |  |
| **3D4/31 cells** |  |  |  |  |  |  |  |
|  | **µM peptide** | | | | | | |
|  | **0.6** | **1.2** | **2.5** | **5** | **10** | **20** | **40** |
| PR-39 | 83 ± 15 | 116 ± 2 | 96 ± 13 | 85 ± 7 | 94 ± 14 | 84 ± 12 | 72 ± 22 |
| PR-39(1-26) | 103 ± 11 | 108 ± 12 | 115 ± 6 | 105 ± 9 | 85 ± 10 | 78 ± 11 | 71 ± 9 |
| PR-39(1-22) | 126 ± 21 | 112 ± 10 | 99 ± 13 | 92 ± 12 | 92 ± 11 | 90 ± 15 | 70 ± 9 |
| PR-39(1-18) | 101 ± 8 | 101 ± 14 | 111 ± 5 | 93 ± 9 | 102 ± 14 | 92 ± 13 | 75 ± 12 |
| PR-39(1-15) | 116 ± 12 | 105 ± 10 | 112 ± 16 | 113 ± 4 | 90 ± 8 | 90 ± 9 | 75 ± 12 |
| PR-39(16-39) | 103 ± 10 | 106 ± 8 | 103 ±b8 | 99 ± 9 | 90 ± 9 | 93 ± 11 | 68 ± 9 |
| PR-39(20-39) | 99 ± 9 | 90 ±9 | 104 ± 7 | 92 ± 15 | 78 ± 6 | 78 ± 13 | 63 ± 13 |
| PR-39(24-39) | 82 ± 9 | 78 ±16 | 78 ± 10 | 80 ± 7 | 66 ± 6 | 72 ± 16 | 57 ± 13 |
